# Regional differences between superficial and deep lumbar multifidus in patients with chronic lumbar spine pathology

**DOI:** 10.1186/s12891-020-03791-4

**Published:** 2020-11-20

**Authors:** Jennifer Padwal, David B. Berry, James C. Hubbard, Vinko Zlomislic, R. Todd Allen, Steven R. Garfin, Samuel R. Ward, Bahar Shahidi

**Affiliations:** 1 Departments of Medicine, University of California, San Diego, USA; 2 Departments of Nanoengineering, University of California, San Diego, USA; 3 Departments of Orthopaedic Surgery, University of California, 9500 Gilman Drive (MC0863), La Jolla, San Diego, CA 92093 USA; 4 Departments of Radiology, University of California, San Diego, USA; 5 Departments of Bioengineering, University of California, San Diego, USA

**Keywords:** Low back pain, Skeletal muscle, Multifidus, Degeneration, Atrophy, Fatty infiltration, Lumbar spine, Lumbar spine pathology, Inflammation, Surgery

## Abstract

**Background:**

Due to its unique arrangement, the deep and superficial fibers of the multifidus may have differential roles for maintaining spine stabilization and lumbar posture; the superficial multifidus is responsible for lumbar extension and the deep multifidus for intersegmental stability. In patients with chronic lumbar spine pathology, muscle activation patterns have been shown to be attenuated or delayed in the deep, but not superficial, multifidus. This has been interpreted as pain differentially influencing the deep region. However, it is unclear if degenerative changes affecting the composition and function of the multifidus differs between the superficial and deep regions, an alternative explanation for these electrophysiological changes. Therefore, the goal of this study was to investigate macrostructural and microstructural differences between the superficial and deep regions of the multifidus muscle in patients with lumbar spine pathology.

**Methods:**

In 16 patients undergoing lumbar spinal surgery for degenerative conditions, multifidus biopsies were acquired at two distinct locations: 1) the most superficial portion of muscle adjacent to the spinous process and 2) approximately 1 cm lateral to the spinous process and deeper at the spinolaminar border of the affected vertebral level. Structural features related to muscle function were histologically compared between these superficial and deep regions, including tissue composition, fat fraction, fiber cross sectional area, fiber type, regeneration, degeneration, vascularity and inflammation.

**Results:**

No significant differences in fat signal fraction, muscle area, fiber cross sectional area, muscle regeneration, muscle degeneration, or vascularization were found between the superficial and deep regions of the multifidus. Total collagen content between the two regions was the same. However, the superficial region of the multifidus was found to have less loose and more dense collagen than the deep region.

**Conclusions:**

The results of our study did not support that the deep region of the multifidus is more degenerated in patients with lumbar spine pathology, as gross degenerative changes in muscle microstructure and macrostructure were the same in the superficial and deep regions of the multifidus. In these patients, the multifidus is not protected in order to maintain mobility and structural stability of the spine.

## Background

In patients with low back pain (LBP) arising from degenerative lumbar conditions, the lumbar multifidus muscle is often observed to have increased fatty infiltration and atrophy compared to similarly aged controls [1], resulting in reduced long-term function and poor prognosis [2–5]. As the multifidus muscle is considered to be a key stabilizer of the lumbar spine [6] – due to its ability to produce high forces over a narrow range of lengths – degenerative changes to the composition and thereby function of this muscle are thought to have profound effects on degenerative lumbar spine disease. This has driven interest in understanding changes in multifidus quality in patients with lumbar spine pathology, in order to determine appropriate treatment strategies to mitigate pathological changes to the multifidus and improve stability of the spinal column [7].

Several studies have investigated microscopic (i.e. cell level) and macroscopic (i.e. whole muscle level) changes in multifidus structure in patients with chronic LBP due to lumbar spine pathology (LSP). Microscopically, type II muscle fiber atrophy, an increased proportion of glycolytic compared to oxidative muscle fibers, decreased vascularity, elevated inflammatory cell count, increased numbers of centrally nucleated fibers, and muscle fiber degeneration has been observed in muscle biopsies obtained from individuals with chronic lumbar spine pathology [8–11]. Macroscopically, pathological changes in the multifidus of patients with LBP such as increases in intramuscular fat infiltration [1, 10, 12, 13] and decreases in cross-sectional area ipsilateral to the side of reported pain [7] have been observed using magnetic resonance imaging (MRI) of the lumbar spine. Qualitatively, intramuscular fat infiltration appears to accumulate in the medial and deep regions of the multifidus in MRI, however regional infiltration of fat in the paraspinal muscles has not been quantified. Given the known effects of chronic lumbar spine pathology on multifidus muscle health, many physical therapy regimens have attempted to reverse structural changes (i.e. hypertrophy, fatty infiltration reversal) in order to restore spinal stability and reduce pain [14]. However, improvements in muscle structure are rarely observed in response to most rehabilitation programs [15–18].

The multifidus muscle is uniquely arranged with superficial fibers spanning two to five levels and located further from the vertebral center of rotation than deep fibers, which cross two levels near the vertebral center of rotation [19–22]. As such, it has been proposed that the larger moment arm of the superficial fibers contributes to extension of the lumbar spine and control of lumbar lordosis [23], whereas the shorter moment arm of the deep fibers contributes to intersegmental compression and stabilization [24]. In addition to mechanically distinct features, differences in muscle activation have been observed using electromyography (EMG) of the superficial and deep multifidus in response to rapid arm movement; the onset of reactive muscle activation in response to arm movement is dependent upon arm swinging direction in the superficial multifidus, but independent of direction in the deep multifidus [25]. This could suggest a more tonic activation pattern for the deep multifidus than the superficial multifidus. In combination, these architectural differences along with observed differences in muscle activation patterns have led to the hypothesis that the superficial and deep multifidus are functionally distinct [22, 26–28]. Similarly, in the presence of LBP, the muscle activation patterns have been shown to be attenuated or delayed in the deep, but not superficial multifidus. This suggests that pain differentially influences the deep region of muscle [29, 30]. As a result, many physical rehabilitation paradigms attempt to specifically target strengthening the deep multifidus in order to restore normal stability and function [31].

While there are different anatomic, histologic, biomechanical, and neuromuscular behavior patterns of the deep and superficial regions of the multifidus, it is unknown if these regions are differentially affected by LSP. Therefore, the purpose of this study was to investigate macrostructural and microstructural differences between the superficial and deep regions of the multifidus muscle in patients with LSP. Specifically, structural features related to muscle function were compared including fat fraction, fiber cross sectional area, fiber type, regeneration, degeneration, vascularity and inflammation. We hypothesized that in a pathologic multifidus muscle, the deep region of the multifidus will exhibit macrostructural and microstructural degeneration compared to the superficial region of the multifidus.

## Methods

### Participants

This was a prospective observational cohort study of 16 patients who underwent lumbar spinal surgery between 2015 and 2016. Patients were included if they were undergoing a primary posterior-approach procedure to address LSP (lumbar stenosis, spondylolisthesis, or disc herniation), and did not have any diagnosed myopathy or systemic neurological condition. All patients provided informed consent for participation in accordance with the declaration of Helsinki and the study protocol was approved by the University of California, San Diego Institutional Review Board.

### Biopsy samples

**Fig. 1:**
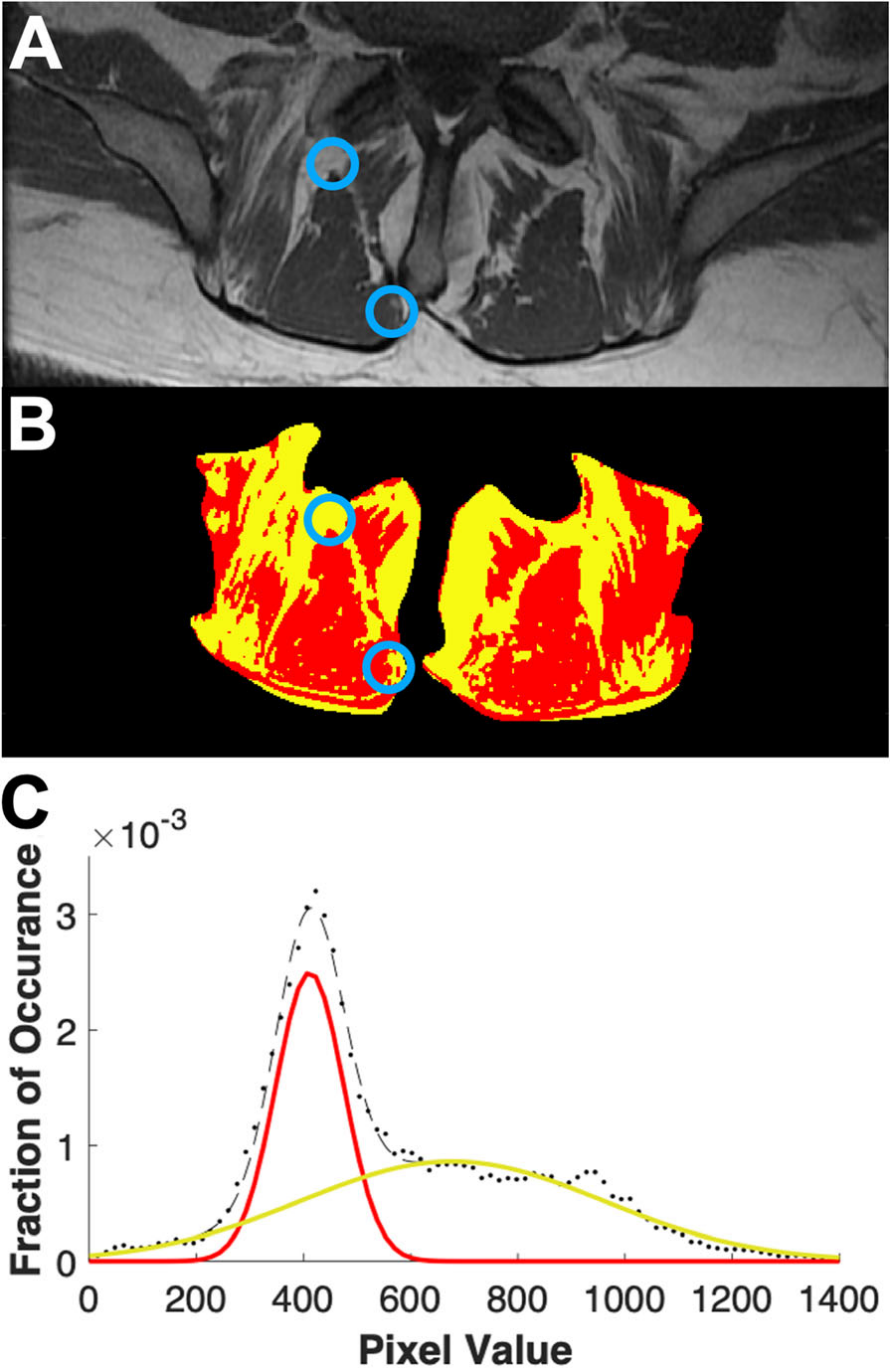
**a** Example MRI used to calculate fat signal fraction. Blue circles indicate approximate location where biopsies were taken from and fat signal fraction measurements were made. **b** Fat signal fraction map of MRI in (**a**). Red indicates voxels classified as muscle, yellow indicates voxels classified as fat. **c** Histogram of pixel intensities in (**a**) across a spectrum of fat (yellow) and muscle (red). Thresholds were defined on a patient-by-patient basis as the intersection between the Gaussian distribution of fat and water

Biopsies (approximately 100 mg) were harvested from the multifidus muscle during surgical approach using a special clamp to keep the tissue at in vivo length [32] at two distinct locations: 1) the most superficial portion of muscle adjacent to the spinous process (designated the “superficial” sample) and 2) approximately 1 cm lateral to the spinous process at the spinolaminar border of the affected vertebral level (designated as the “deep” sample) (Fig. 1). Each surgeon was trained by the same research staff on the appropriate location of the biopsy and researchers verified the biopsy location for each procedure. Biopsies were excised upon initial approach in order to maximize anatomical consistency and avoid the influence of retraction. Biopsies were immediately pinned at in vivo length and flash frozen in liquid nitrogen cooled isopentane [32]. Frozen samples were transported back to the laboratory on dry ice and were stored at − 80 °C until processing. Samples were embedded in optimal cutting temperature compound, and cut into 10 μm sections using a cryostat (Leica CM3050S, Buffalo Grove, IL).

**Fig. 2:**
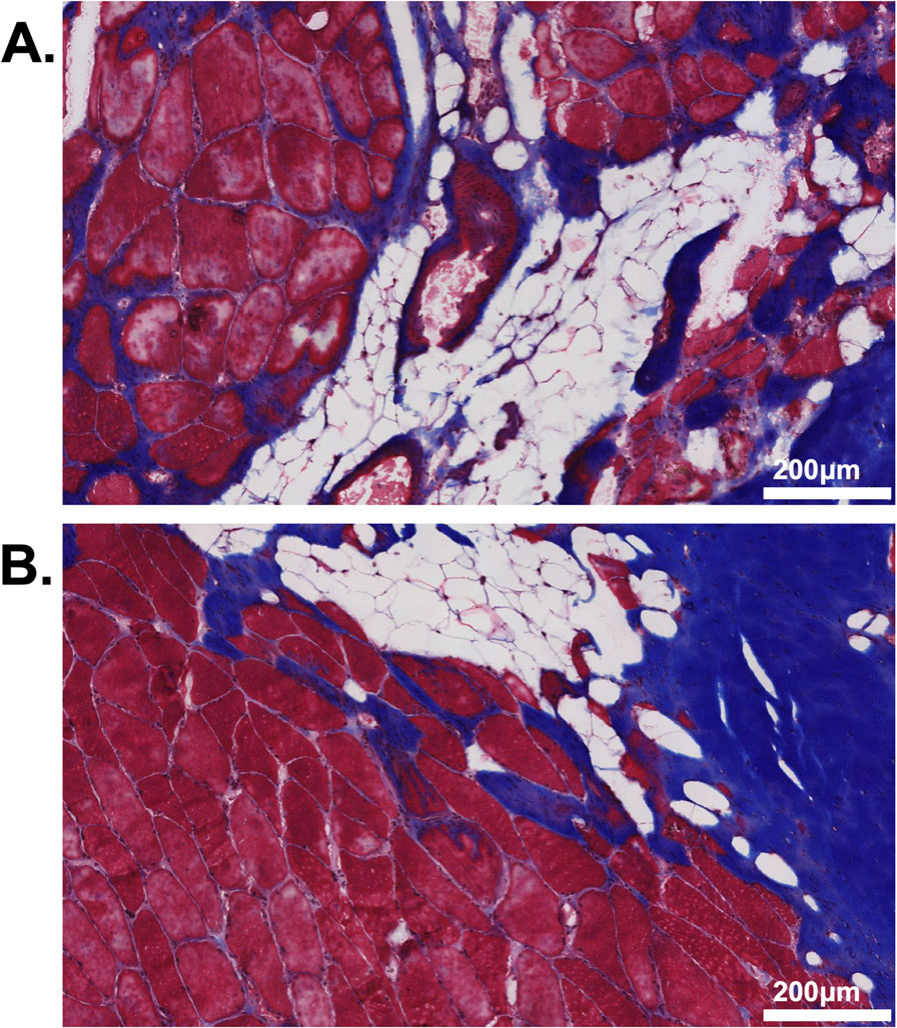
Gomori trichrome stained biopsy section demonstrating regions of muscle, collagen, and fat from superficial (**a**) and deep (**b**) multifidus biopsies

Tissue morphology and composition were visualized using Hematoxylin and Eosin (H&E) and Gomori Trichrome stains (Fig. 2) [33]. Relative fractions of muscle, fat, and collagen were quantified from Trichrome stained biopsies using ImageJ software [34]. Manual intensity thresholding was used for tissue type segmentation between red (muscle), green (loose collagen), and blue (dense collagen) channels of whole section slides. Adipose tissue was identified morphologically and manually traced. To quantify muscle fiber area and centrally nucleated fibers, samples were stained with Laminin-111 or Laminin-211 to identify muscle basal laminar borders (LAMA1 (Sigma L9393) or LAMA2 (Vector VP-M648)), Type I (Hybridoma Bank BA-D5) and IIa (Hybridoma Bank SC-71) Myosin Heavy Chain antibodies to identify fiber type, counterstained with 4′6-diamidino-2-phenylindole (DAPI) to identify cell nuclei, and then coverslipped with Vectashield mounting medium (Vector H-5000). Six randomly generated frames from biopsy cross-sections were used to quantify fiber area and centrally nucleated fibers (a measure of regeneration/degeneration) by counting cells with DAPI signal within the muscle fiber area. Vascularity was measured by counting the number of Von Willebrand Factor (Sigma F3520) positive vessels/mm^2^, [35]. Muscle regenerative capacity was measured by calculating the number of Pax7^+^ satellite cells per muscle fiber from 10 randomly generated 20x fields. Muscle degeneration was quantified by manually evaluating the proportion of 1 mm^2^ grids across an entire section of muscle that contained degenerative muscle fibers including: moth eaten fibers, cell infiltration, core fibers, and myophagocytosis as previously described [36].

### MRI measurements

Preoperative MRIs from 15 biopsied patients were obtained from our hospital database (1 patient had no preoperative MRI available). Regions of interest (ROI) measuring 1 cm^2^ were isolated from T1-weighted axial magnetic resonances images taken at a single slice at the vertebral level and side that the biopsies were taken (Fig. 1a, b). A custom written Matlab (Mathworks, Natick MA) code was used to measure the fat signal fraction (FSF) within the ROI [1, 37]. Pixels within the ROI were identified as either fat or muscle based on pixel intensity, where a two term gaussian model was fit to the histogram of pixel intensities and the threshold was set at the intersection of the gaussian distributions. Pixel values below the threshold were classified as muscle and pixels above were classified as fat (Fig. 1c) [1, 37].

### Statistical analysis

Paired t-tests were used to evaluate the difference in histologic- (tissue composition, fiber area (by fiber type), centralized nuclei, vascularity, degeneration) and MRI-based (fat signal fraction) measures of muscle health between the superficial and deep regions of the multifidus. The threshold for statistical significance (α) was set to 0.05 for all analyses. Effect size (Cohens d [38]) was calculated for all statistical tests. Data are reported as mean ± standard deviation. Statistical analyses were performed using Prism 8 (GraphPad, La Jolla, CA).

## Results

The average age of participants was 61.9 years ±17.3 years with 6 female patients. The average duration of symptoms in these patients was 3.8 years ±6.1 years. Surgeries were performed for a diagnosis of lumbar stenosis in 12 patients, spondylolisthesis in 2 patients, and disc herniation in 2 patients. There was an equal distribution between biopsies taken on the L vs R side (Table 1). Effect size (d) for all comparisons is reported in Table 2.

**Table 1:** Demographics of patients included in this study

Age (years)	61.9 ± 17.3
Gender (M:F)	10:6
Duration of Symptoms (years)	3.8 ± 6.1
Biopsy Side (L:R)	8:8
Biopsy Level	
L3	5
L3-L4	4
L4	1
L4-L5	5
L5	0
L5-S1	1
NPRS (points)	5.7 ± 2.5
ODI (%)	47.6 ± 17.9

*NPRS* numeric pain rating scale, *ODI* Oswestry disability index

**Table 2:** Cohen’s effect size (d) analysis for all statistical comparisons

Measurement	Effect size
Area fraction fat	0.11
Area fraction muscle	0.34
Area fraction collagen	0.14
Dense collagen	0.64
Loose collagen	0.85
Type I fiber area	0.01
Type II fiber area	0.33
Distribution type I	0.04
Distribution type IIa	0.13
Distribution type IIx	0.09
% centralized nuclei	0.21
% Pax7^+^	0.29
% von Willebrand factor^+^	0.69
Degeneration	0.20
MRI fat signal fraction	0.04

### Histologic differences between superficial and deep biopsies of the multifidus muscle

**Fig. 3:**
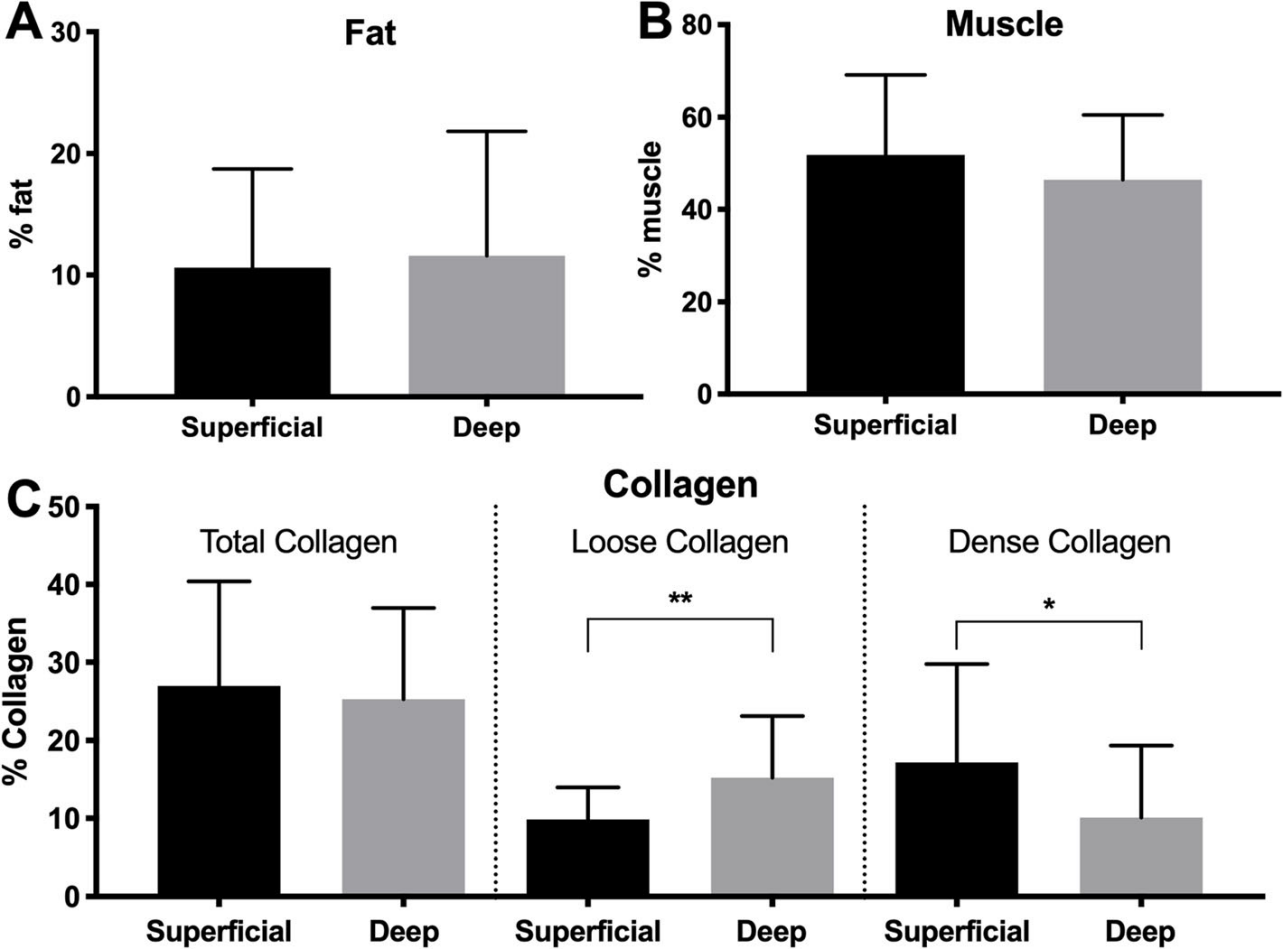
Histologic evaluation of tissue composition. Area fraction of fat (**a**), muscle (**b**), and collagen (**c**) of superficial (black) and deep (gray) biopsy samples. * - *p* < 0.05; ** - *p* < 0.01

**Fig. 4:**
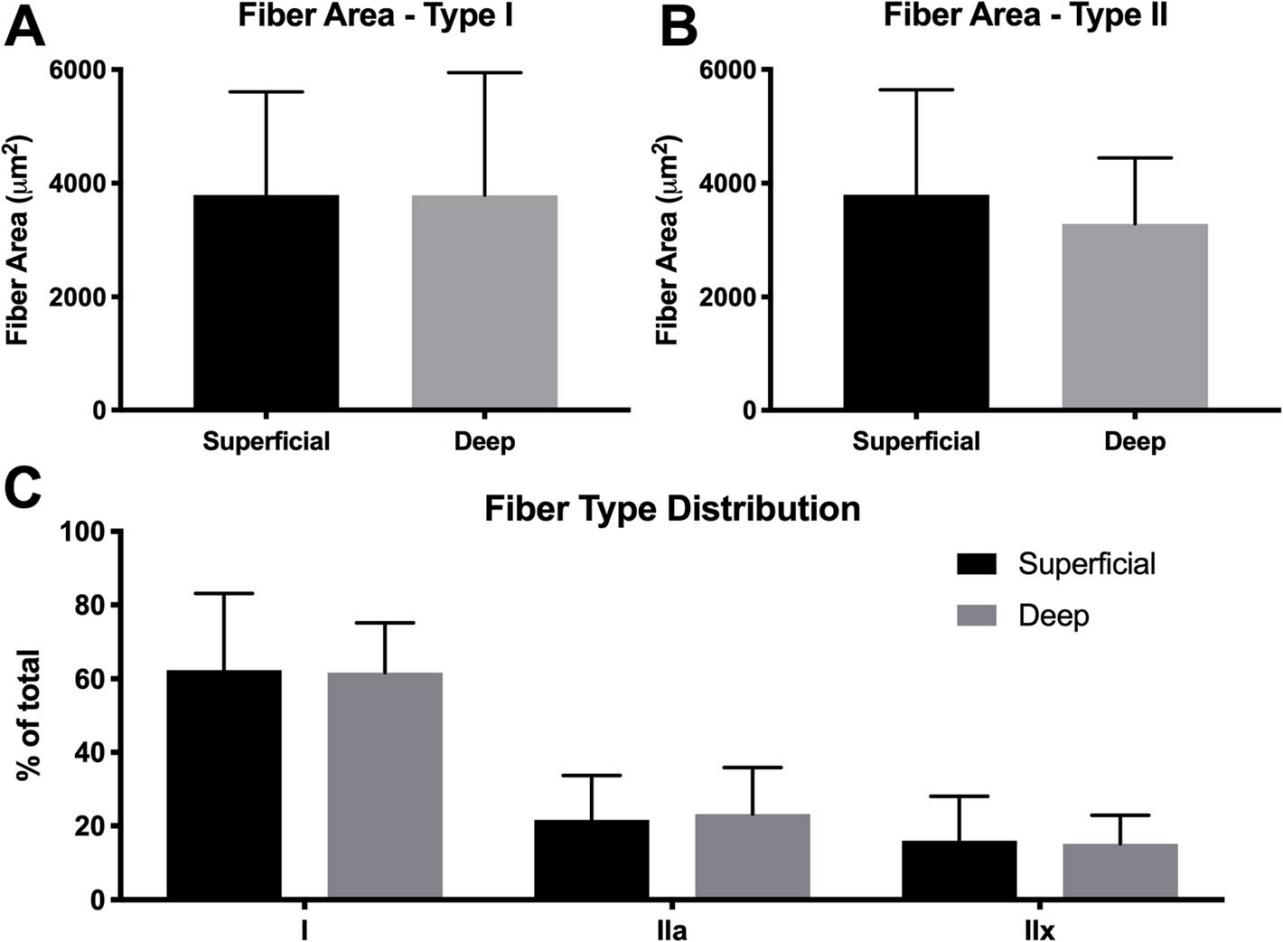
Fiber area of type I (**a**) and type II (**b**) muscle fibers for superficial (black) and deep (gray) biopsy samples. **c** Distribution of fiber types between superficial (black) and deep (gray) biopsy samples

Overall, biopsy samples were composed of 11.1% ± 9.1% fat, 49.2% ± 15.7% muscle, and 26.2% ± 12.4% collagen. No significant difference was found for the area fraction of fat (*p* = 0.67, d = 0.10), muscle (*p* = 0.16, d = 0.34), or total collagen (*p* = 0.59, d = 0.14) between superficial and deep samples (Fig. 3a, b). However, superficial biopsies were found to have a greater average proportion of dense collagen (17.18% vs 10.09%, *p* = 0.034, d = 0.42) and smaller average proportion of loose collagen (9.86% vs 15.23%, *p* < 0.01, d = 0.64) compared to deep biopsies (Fig. 3c). No significant difference in either type I (*p* = 0.77, d = 0.05) or type II (*p* = 0.40, d = 0.01) fiber area was found between superficial and deep samples (Fig. 4a, b). Additionally, there was no significant difference in the distribution of type I (*p* = 0.97, d = 0.04), type IIa (*p* = 0.95, d = 0.13), and type IIx (*p* = 0.90, d = 0.09) fibers between superficial and deep samples (Fig. 4c).

**Fig. 5:**
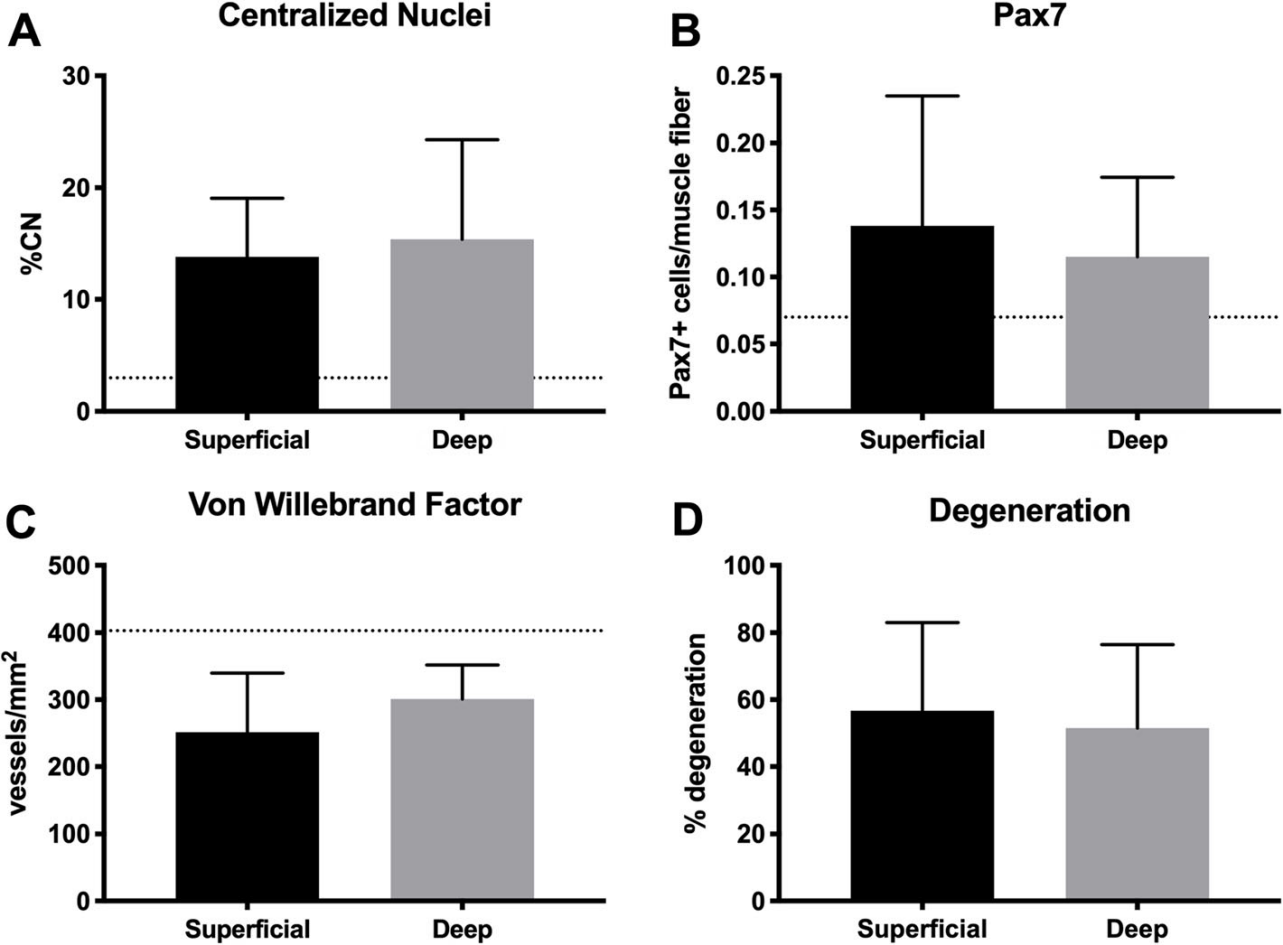
Histologic measurements of muscle regeneration, vascularization, and degeneration. Muscle regeneration was defined by the percentage of fibers with centralized nuclei (**a**) and Pax7^+^ cells per muscle fiber (**b**), demonstrating elevation compared to prior literature in muscle [39, 40]. Vascularization was assessed by that number of Von Willebrand Factor positive vessels per square millimeter (**c**) demonstrating lower vascularity compared to prior literature in muscle [41]. Degeneration was quantified by the percentage of muscle fibers that exhibited signs of degeneration (moth eaten fibers, cell infiltration, core fibers, and myophagocytosis) per square millimeter. Measurements were made for both superficial (black) and deep (gray) biopsy samples

Histologic markers of muscle regeneration and degeneration were increased, and vascularity was decreased compared to literature based reports from normal muscle (Fig. 5) [39–41]. However, no significant difference was found between superficial and deep multifidus biopsies for fraction of fibers with centralized nuclei (*p* = 0.85, d = 0.21) or Pax7^+^ cells (*p* = 0.70, d = 0.29), von Willebrand factor + vessels/area (*p* = 0.18, 0.69), or histologic evidence of muscle degeneration (*p* = 0.35, d = 0.20).

### MRI measured differences between superficial and deep fat fraction of the multfidus muscle

**Fig. 6:**
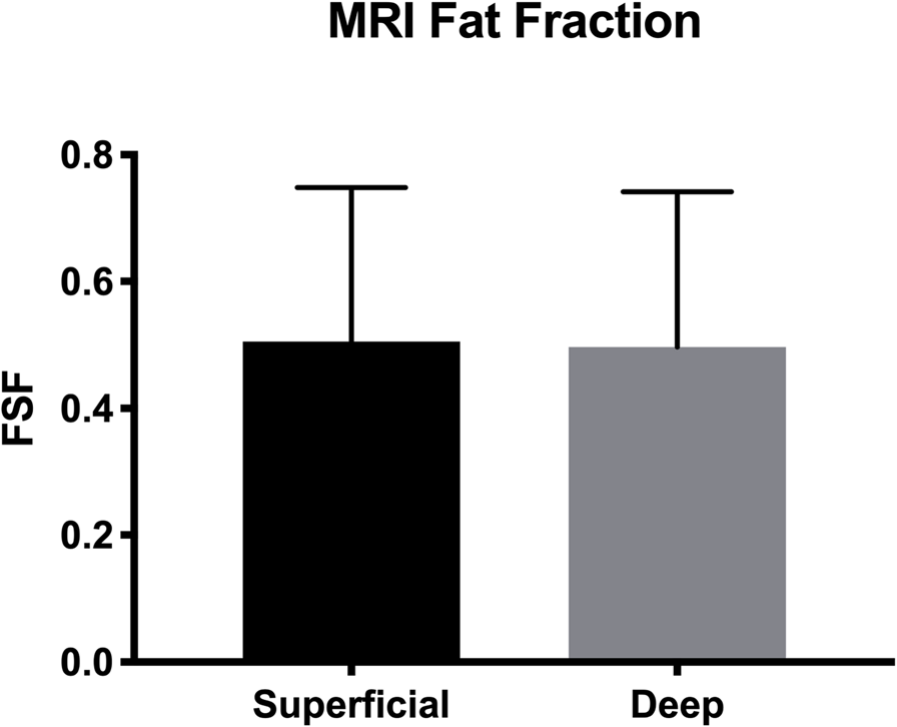
MRI measured fat signal fraction of the superficial (black) and deep (gray) regions of interest that corresponded to the approximate locations of where the multifidus was biopsied

Qualitatively, fatty infiltration of the posterior musculature was observed along the spinolaminar border, between the multifidus and erector spinae muscles, and in the posterior epimuscular fatty region of the erector spinae. As both the superficial and deep biopsy regions reside in regions of high fat content, it was not surprising that these areas quantitatively had high fat signal fractions. However, no significant difference in MRI-assessed fat signal fraction was found between superficial and deep regions of interest coincident with where muscle biopsies were taken (*p* = 0.92, d = 0.05; Fig. 6).

## Discussion

Contrary to our hypothesis, there were no significant differences between the superficial and deep regions of the multifidus for histologically measured area of fat, muscle area, fiber cross sectional area, muscle regeneration markers, muscle degeneration markers, and vascularization. While total collagen content between the two regions was the same, the superficial region of the multifidus was found to have less loose and more dense collagen than the deep region. These findings suggest that the functional differences that have been previously observed between the superficial and deep regions of the multifidus are not a result of muscle composition, and that muscle degeneration does not occur preferentially in a given region of the multifidus.

Differences in paraspinal muscle activation during motion and muscle composition have been highlighted as targets in assessing both muscle health and potential for improvement with physical therapy [22, 42]. Lumbar multifidus has been especially implicated given its assumed role in spinal stability and common pathological changes in LBP [43–47]. The role of the multifidus has further been parsed out based on its distinct superficial and deep layers, with the superficial multifidus thought to be involved primarily in lumbar extension and the deep multifidus focused on control of dynamic intersegmental stability via intervertebral compression, thus providing spinal stability [22, 25, 48]. Given these differences in activation patterns, it has previously been hypothesized that there would be differences in proportion of muscle fiber types between layers. Specifically, the deep multifidus would have a higher proportion of type 1 fibers compared to the superficial multifidus [22, 48], since type I fibers are fatigue resistant and most suitable to providing the constant low load activity necessary to provide dynamic lumbar stability. Importantly, this supposedly higher proportion of type I fibers in deep multifidus has been used as a target for the low load, tonic exercises often implemented in LBP exercise rehabilitation regimens [22, 49]. Studies comparing the proportion of these fibers in patients have demonstrated conflicting results. One study examining both autopsy specimens and biopsies from patients undergoing surgery for disc herniation found a significantly greater proportion of type I fibers in deep multifidus [50]. However, the majority of the literature has demonstrated no difference in fiber-type distribution based on multifidus depth across a range of ages, consistent with the results in this study [8, 9, 51–55].

Regional differences in collagen within the lumbar multifidus have not been thoroughly explored. Our histological results found that although the superfical and deep muscle biopsies both had an elevated area fraction of collagen (~ 25%) and superficial mutifidus biopsies had less dense collagen and more loose collagen than deep multifidus biopsies. Increased collagen expression within muscle tissue has been associated with chronic inflammatory changes and subsequent irregular remodeling thought to be related to LSP [56]. Such changes often accompany muscle atrophy and increased fat deposition to comprise what is defined as tissue fibrosis. Progression of paraspinal muscle fibrosis is associated with decline in muscle functionality and recovery at these late stages is prolonged. Previous animal studies examining thoracolumbar fascia (TLF) in rats have shown an organized distribution of collagen based on tissue depth, with more superficial layers having a higher percentage of densely packed collagen fibers compared to deeper layers of the TLF, which were composed of loose, irregularly oriented collagen fibers [57]. Increases in connective tissue within multifidus appear to be related to duration of LBP, with injury models only demonstrating increases in collagen area after several months [14]. Additionally, fibrotic gene expression has been shown to be upregulated in individuals with chronic symptoms compared to those with acute symptoms [58]. These results suggest that fibrotic deposition within muscle may affect muscle function in patients with LSP, further predisposing patients to maladaptive muscle changes and fatty infiltration. The mechanisms underlying the increase in collagen deposition in multifidus are not fully understood at this time, but physical activity appears to reduce the inflammation associated with this tissue fibrosis, which may improve whole muscle function [56, 59–64].

Markers of muscle vascularity, damage, and degeneration did not significantly differ between superficial and deep samples Although the average percentage of centralized nuclei for each biopsy level was higher than expected for healthy skeletal muscle (3%), this percentage was lower than values reported for other studies examining lumbar spine pathology [10, 11]. Despite the high proportion of markers for muscle recovery (centralized nuclei for regeneration and Pax7+ for satellite cell density), the number of muscle fibers that exhibited degenerative characteristics remained high. This is consistent with previous results from our group that suggest the amount of muscle degeneration in chronic LBP patients outpaces the ability to restore a healthy muscle phenotype [10, 65].

Although this study provides the first documented comparison between superficial versus deep histology and MR imaging of the lumbar multifidus in patients with LSP, there were several limitations that must be considered in assessing our results. First, the nature of biopsy collection was such that identifying and matching the exact corresponding region to the MRI was not possible. We defined an ROI on MRI as close to the biopsy location as possible, based on a standardized biopsy region in our protocol and biopsy level identification via intraoperative fluoroscopy. By involving a limited number orthopedic surgeons and training them on biopsy technique and location, we attempted to mitigate variability in biopsy location. Second, it is impossible to measure collagen from routine clinical imaging (i.e. T1-weighted, T2-weighted MRIs) due to the short transverse relaxation time of fibrotic tissues. While MRI pulse sequenceses sensitive to collagen in tissues exist - such as ultrashort echo time - they are not routinely used to assess patients with LSP. Third, this was a prospective study and only 16 patients were included. However from this sample population, it was unlikely that differences in muscle microstructure/macrostructure between the superficial and deep multifidus would be evident with a larger sample size. Fourth, the majority of patients in this study all had an identifiable lumbar spine pathology such as stenosis or disc degeneration resulting in radicular symptoms, which may differentially impact superficial and deep regions of the multifidus as compared to nonspecific LBP. While biopsies from subjects with nonspecific LBP would allow for the differentiation of histological changes as a result of potential denervation, this patient population is less likely to receive surgical intervention without an identified anatomical pain generator and ethical issues often preclude obtaining regional biopsies. Furthermore, this study did not take into account some patient factors that have been implicated in muscle quality, such as spinal alignment [66, 67], or level of physical activity [56, 59].

## Conclusions

The results of our study did not support the hypothesis that the deep region of the multifidus is more degenerated in patients with lumbar spine pathology, as gross degenerative changes in muscle microstructure and macrostructure were the same in the superficial and deep regions of the multifidus. Although total collagen deposition was similar between regions, we found that superficial biopsies of the muscle demonstrated a greater percent dense collagen and lower percent loose collagen compared to deep biopsies. However, the functional implications of differences in the distribution of loose and dense collagen are unclear. More studies are warranted to further elucidate functional contribution of loose and dense collagen deposition on multifidus passive mechanics in patients with degenerative lumbar spine conditions.

## Data Availability

The datasets used and/or analyzed during the current study are available from the corresponding author on reasonable request.

## Abbreviations

DAPI: 4′6-diamidino-2-phenylindole
H&E: Haematoxlyn & eosin
LBP: Low back pain
LSP: Lumbar spine pathology
MRI: Magnetic resonance imaging
ROI: Region of interest
TLF: Thoracolumbar fascia
